# Food patterns in relation to weight change and incidence of type 2 diabetes, coronary events and stroke in the Malmö Diet and Cancer cohort

**DOI:** 10.1007/s00394-018-1727-9

**Published:** 2018-05-31

**Authors:** Ulrika Ericson, Louise Brunkwall, Joana Alves Dias, Isabel Drake, Sophie Hellstrand, Bo Gullberg, Emily Sonestedt, Peter M. Nilsson, Elisabet Wirfält, Marju Orho-Melander

**Affiliations:** 1 Department of Clinical Sciences in Malmö, Diabetes and Cardiovascular Disease, Genetic Epidemiology, Lund University, Malmö, Sweden; 2 Department of Clinical Sciences in Malmö, Nutritional Epidemiology, Lund University, Malmö, Sweden; 3 Department of Internal Medicine, Skåne University Hospital, Lund University, Malmö, Sweden; 4 Clinical Research Centre, building 60, floor 13, SUS in Malmö, entrance 72, Jan Waldenströms gata 35, 205 02 Malmö, Sweden

**Keywords:** Food intake, Epidemiology, Weight gain, Type 2 diabetes, Cardiovascular diseases

## Abstract

**Purpose:**

We examined if data-driven food-patterns associate with weight change, incidence of type 2 diabetes (T2D), coronary events (CE) and stroke.

**Methods:**

The study included 20,487 individuals (61% women) from the Malmö Diet and Cancer cohort, 45–74 years, without diabetes and CVD at baseline (1991–1996) and who did not report dietary changes. Diet was measured with a modified diet history method. During 15 years follow-up, 2206 T2D, 1571 CE and 1332 stroke cases were identified. Data on weight change after 16.7 years were available in 2627 individuals.

**Results:**

From principal component analysis, we identified six food-patterns which were similar in women and men. The first pattern, explaining 7% of the variance, was characterized by high intake of fibre-rich bread, breakfast cereals, fruits, vegetables, fish and low-fat yoghurt, and by low intake of low-fibre bread. This health conscious pattern was associated with lower T2D risk (HR comparing highest quintile with lowest: 0.75; 95% CI 0.61–0.92, 0.82; 95% CI 0.68–1.00 in women and men, respectively, *P* trends = 0.003, 0.01) and CE (HR 0.77; 95% CI 0.58–1.02, HR 0.83; 95% CI 0.68–1.01, *P* trends = 0.05, 0.07), and in men also with lower risk of ischemic stroke (HR 0.69; 95% CI 0.54–0.88; *P* trend = 0.001) and less pronounced weight gain (0.93 kg/10 years, *P* trend = 0.03). A low-fat product pattern was associated with increased T2D risk in gender combined analyses (*P* trend = 0.03) and a pattern characterized by dressing and vegetables with lower CE risk in men (*P* trend = 0.02).

**Conclusions:**

Our main finding was that a dietary pattern indicating health conscious food choices was associated with lower risk of cardiometabolic diseases in both genders.

**Electronic supplementary material:**

The online version of this article (10.1007/s00394-018-1727-9) contains supplementary material, which is available to authorized users.

## Introduction

The prevalence of obesity, type 2 diabetes (T2D) and cardiovascular disease is increasing globally. Although many lifestyle-related risk factors have been identified, the great challenge of designing public health recommendations, including dietary advice to stop this negative trend, still remains.

Diet is an extremely complex exposure and during the last decades, the value of examining intake of foods or food patterns, in addition to nutrients, has been highlighted [1]. Correlations and interactions between food components may not be satisfactorily taken into account in observational studies on single dietary components [1], which complicates the interpretation of findings in nutritional epidemiology. It is difficult to isolate the effect of specific nutrients or foods. In addition, cumulative effects of several food components may be of greater magnitude and, therefore, easier to detect [2]. Moreover, we do not eat single nutrients or foods, but combinations of these, and it is, therefore, of great value to capture overall dietary patterns that could be translated into relevant food-based dietary guidelines. One option is to use data-driven statistical methods to reduce intake data on a number of single foods or food groups into meaningful eating patterns reflecting how foods are commonly consumed together.

Several so-called “healthy/prudent” data-driven dietary patterns have been found to associate with lower risk of T2D [3, 4] and cardiovascular disease [5], whereas “unhealthy/western” patterns seem to associate with increased risk of T2D [4]. A few studies have also derived dietary patterns associated with weight change [6–9]. Still, it is important to examine dietary patterns in different populations, as the food types, food preparation, as well as how foods are combined differ and observed findings in one study may, therefore, not be applicable to food-based guidelines in other populations.

In this population-based prospective study of men and women from the Swedish Malmö Diet and Cancer (MDC) cohort, we aimed to identify food patterns using principal component analysis and to examine associations with weight change, and incidence of T2D, coronary disease and stroke.

## Subjects and methods

### Study population and data collection

The MDC study is a population-based prospective cohort study in Malmö, a city in the south of Sweden. Baseline examinations were conducted between 1991 and 1996. All women born 1923–1950 and all men born 1923–1945, living in the city of Malmö, were invited to participate (*n* = 74,138). Details of the cohort and the recruitment procedures are described elsewhere [10]. The only exclusion criteria were mental incapacity and inadequate Swedish language skills (eligible persons = 68,905). The participants filled out questionnaires covering socio-economic, lifestyle and dietary factors, and recorded meals, and underwent a diet history interview. Anthropometric measurements were conducted by nurses. Weight was measured using a balance-beam scale with subjects wearing light clothing and no shoes. Standing height was measured with a fixed stadiometer calibrated in centimeters. Waist circumference was measured midway between the lowest rib margin and the iliac crest. Body composition was estimated with a bioelectrical impedance analyzer (BIA 103, RJL systems, single-frequency analyzer, Detroit, USA). Body fat percent was calculated using an algorithm provided by the manufacturer. During the screening period, 28,098 participants (40% of the eligible persons) completed all baseline examinations. In the current study, we included 20,487 participants (12,456 women and 8031 men) free of diabetes and cardiovascular disease at baseline and who did not report previous dietary changes (Supplementary Fig. 1). We excluded 1193 individuals, based on self-reported diabetes diagnosis, self-reported diabetes medication or information from medical data registries (see below) indicating a date of diagnosis preceding baseline examination date and 855 individuals with a history of coronary events or stroke. Among those without history of diabetes, coronary events and stroke, we excluded 5149 individuals who reported dietary change in the past based on the question “Have you substantially changed your eating habits because of illness or some other reasons?”, because intake changes of specific foods may change the relative intake of other foods and thereby the overall dietary patterns. Among the 20,487 individuals in this study, 2627 could be included in the analysis of longitudinal weight change, as they participated in follow-up examinations between 2007 and 2012. For the follow-up examination, all individuals that were alive and still living in Sweden (*n* = 4924) from a random sub-cohort of 6103 MDC participants, the MDC cardiovascular Cohort, were invited to participate. The ethical committee at Lund University has approved the study (LU 51–90) and the participants have given their written informed consent.

### Dietary data

Dietary data were collected once during the baseline period. The MDC study used an interview-based, modified diet history method that combined (1) a 7-day menu book (food record of meals that vary from day to day) (usually lunch and dinner meals), cold beverages and nutrient supplements, and (2) a 168-item food frequency questionnaire for assessment of consumption frequencies and portion sizes of regularly eaten foods that were not covered by the 7-day menu book. Finally, (3) a 45-min interview completed the dietary assessment. The MDC method is described in detail elsewhere [11, 12].

The diet analyses were adjusted for a variable called “diet method version” because slightly altered coding routines of dietary data were introduced in September 1994 to shorten the interview time (from 60 to 45 min). The altered coding routines resulted in two slightly different method versions (before or after September 1994) without any major influence on the ranking of individuals [12].

The relative validity of the MDC method was evaluated in the Malmö Food study 1984–1985, comparing the method with 18 days weighed food records [13, 14]. The Pearson correlation coefficients, adjusted for total energy, between the reference method and the MDC-method, were in women and men, respectively, for intakes of bread 0.58/0.50, cereals 0.73/0.74, fruits 0.77/0.60, vegetables 0.53/0.65, low-fat milk 0.92/0.90, high-fat milk 0.75/0.76, cheese 0.59/0.47, fish 0.70/0.35, low-fat meat 0.51/0.43 and for high-fat meat 0.80/0.40 [13].

The mean daily intake of foods was calculated based on frequency and portion size estimates from the questionnaire and menu-book. The food intake was converted to energy and nutrient intakes using the MDC nutrient database where the majority of the nutrient information comes from PC-KOST2-93 from the National Food Agency in Uppsala, Sweden. The food intakes were aggregated into 33 groups to obtain food groups more frequently consumed in the population, but to keep characteristics related to both dietary behaviors and nutrient content. When aggregating the foods, some special concern was taken regarding fat and fibre contents, as the dietary assessment was especially designed to capture those intakes [15] and as dietary fat and fibre are thought to be crucial in the development of cardiometabolic disease [16, 17]. Energy-adjusted intakes of the 33 food groups were obtained by regressing the intakes on non-alcohol energy intake.

### Ascertainment of diabetes, coronary events and stroke

We identified 2206 incident cases of T2D during 304,182 person years of follow-up via at least one of seven registries (90%) or at new screenings or examinations during follow-up (10%). The mean follow-up time was 15 years (range 0–20). In total, 1571 coronary event cases and 1332 stroke cases were identified during 312,262–312,303 person years of follow-up, respectively, with a mean follow-up time of 15 years (range 0–20). The subjects contributed person-time from date of enrolment until date of diagnosis, death, migration from Sweden, or end of follow-up (December 2010), whichever occurred first. During follow-up 0.5% had migrated from Sweden.

If available, we used information on the date of T2D diagnosis from two registries prioritized in the following order: (1) the regional Diabetes 2000 registry of Scania [18] and (2) the Swedish National Diabetes Registry [19]. These registries required a physician diagnosis according to established diagnosis criteria (fasting plasma glucose concentration ≥ 7.0 mmol/L or fasting whole blood concentration ≥ 6.1 mmol/L, measured at two different occasions). Individuals with at least two HbA1c values above 6.0% with the Swedish Mono-S standardization system (corresponding to 6.9% in the US National Glycohemoglobin Standardization Program and 52 mmol/mol with the International Federation of Clinical Chemistry and Laboratory Medicine (IFCC) units) [20, 21] were categorized as diabetes cases in the Malmö HbA1c Registry. In addition, cases were identified via four registries from the National Board of Health and Welfare in Sweden: the Swedish National Inpatient Registry, the Swedish Hospital-based outpatient care, the Cause-of-death Registry and the Swedish Prescribed Drug Registry. Type of diabetes was based on the glycaemic parameters, treatment/medication, age at diagnosis, GADA, C-peptide and BMI.

Information about prevalent and incident coronary event and ischemic stroke was taken from the national Swedish Hospital Discharge register, Cause-of-death register [22] and the local stroke register in Malmö (STROMA) [23]. A coronary event was defined on the basis of codes 410–414 (fatal or non-fatal myocardial infarction or death due to ischemic heart disease) in the International Classification of Diseases, 9th Revision (ICD-9). Ischemic stroke was defined based on code 434 (ICD-9) and diagnosed when computed tomography, magnetic resonance imaging or autopsy could verify the infarction and/or exclude haemorrhage and non-vascular disease. If neither imaging nor autopsy was performed, the stroke was classified as unspecified. Haemorrhagic or non-specific stroke cases (ICD-9 code 430, 431 and 436) were excluded since these subtypes of stroke do not have the same underlying risk factors as ischemic stroke. National Tax Board provided information on vital status and emigration.

### Weight change

Weight was measured both at baseline and at follow-up examinations in 2627 individuals, with on average 16.7 years between the examinations. The yearly weight change was calculated: *weight at follow-up* minus *weight at baseline* divided by *number of follow-up years*. Mean 10-year weight change was obtained by multiplying the yearly weight change by 10.

### Other variables

Information on age was obtained from the personal identification number. Body mass index (BMI; kg/m^2^) was calculated from direct measurement of weight and height. Leisure time physical activity was assessed by asking the participants to estimate the number of minutes per week they spent on 17 different activities. The duration was multiplied with an activity specific intensity coefficient and an overall leisure time physical activity score was created. The score was divided into gender-specific quintiles. The smoking status of the participants was defined as current smokers (including irregular smokers), ex-smokers and never-smokers. The total consumption of alcohol was defined by a four-category variable. Participants reporting zero consumption in the menu book, and indicating no consumption of any type of alcohol during the previous year, were categorized as zero-reporters. The other category ranges were < 15 g alcohol/day for women and < 20 g/day for men (low), 15–30 g/day for women and 20–40 g/day for men (medium), and > 30 g/day for women and > 40 g/day for men (high). Participants were divided into four categories according to their highest level of education (≤ 8, 9–10, 11–13 years or university degree). Season was defined as season of diet data collection (winter, spring, summer and fall).

### Statistical analysis

The SPSS statistical computer package (version 24.0; IBM Corporation, Armonk, NY, USA) was used for all statistical analyses. All food variables were log transformed (e-log) to normalize the distribution before analysis. To handle log transformation of zero intakes, we added a very small amount (0.01 g). All food intakes were energy adjusted with the residual method.

We used principal component analysis (eigenvalues > 1 and varimax rotation) to reduce 33 energy-adjusted food groups into factors representing food patterns. We derived factors separately in women and men. From the obtained Scree plots (Supplementary Figs. 2a and 2b), we decided to retain and rotate the six factors (eigenvalues > 1.2) that explained most of the variance in the data in both genders. These factors were possible to interpret and translate into food patterns based on their loadings for the initial food group variables. Reported characteristics of the patterns were based on food group loadings < − 0.25 and > + 0.25.

We examined baseline characteristics across quintiles of the factors representing food patterns with the general linear model for continuous variables (adjusted for age) and with Chi square test for categorical variables. We used Cox proportional hazards regression model to estimate hazard ratios (HRs) of incident T2D, coronary events and stroke associated with quintiles of factors representing food patterns. The first quintile was used as the reference. Years of follow-up was used as the underlying time variable. To assess the proportional hazards assumption, we used graphs and tested interactions between the underlying time variable and examined covariates. The assumption was considered to be satisfied for all covariates except age with regard to T2D in women. We, therefore, additionally performed T2D-analyses with age-stratified cox models (per 1-year age interval) in women, but the results remained unchanged. The general linear model was used to examine mean 10-year weight change in quintiles of the food patterns. If non-significant tendencies of associations in the same direction were seen in both genders (no statistical interaction with gender), we performed gender-combined analyses on factors representing similar food patterns in both genders. Significant observations from gender-combined analyses are reported in the text.

We used covariates obtained from the baseline examinations. A basic model included adjustments for age (continuous), diet method version, season (categorical) and total energy intake (continuous). Our full multivariable model additionally included adjustments for the following categorical variables: leisure time physical activity, smoking, alcohol intake, and education, and finally baseline BMI as a continuous variable. Since associations between diet and cardiometabolic disease may partly be mediated via BMI, we also performed analyses with an intermediate multivariable model without inclusion of BMI. The covariates were identified from the literature and indicated potential confounding in the MDC cohort, due to associations with incident cardiometabolic diseases and dietary intakes. We also made additional adjustments for waist circumference. In analyses of weight change, we also performed analyses with adjustment for baseline weight. Missing values for the variables were treated as separate categories. Tests for interactions between the food patterns and obesity status or sex with regard to the incident diseases were performed [e.g. BMI (≤ 25 or > 25) × quintile of food patterns (treated as continuous variables)]. In sensitivity analyses, we excluded individuals above 60 years of age at baseline in analyses of T2D, coronary events and stroke, as these participants could be regarded as elderly already at baseline. Although they did not report to have made any major dietary changes, information about their diet, physical activity and weight may not reflect that of their previous life. All statistical tests were two-sided and statistical significance was assumed at *P* < 0.05.

## Results

### Food patterns

**Fig. 1:**
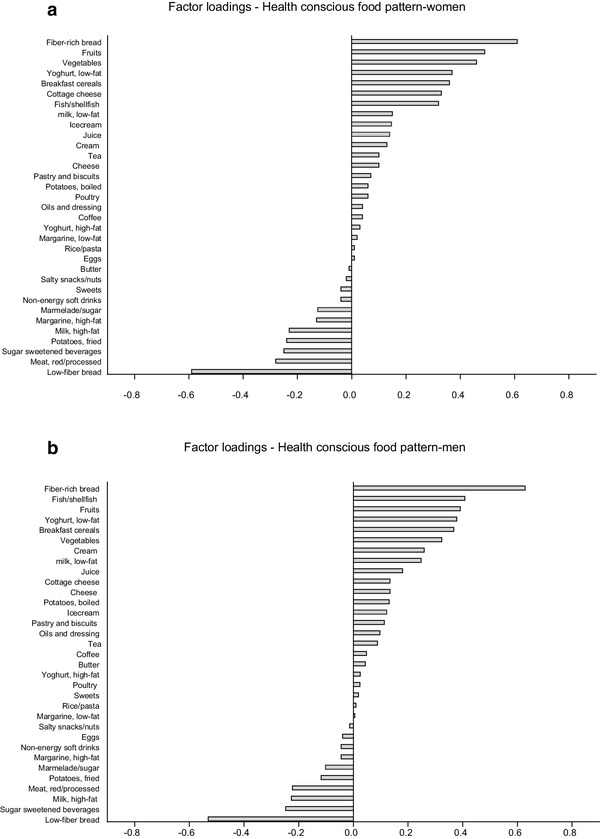
**a** The food pattern that explained most of the variance in women (7%). **b** The food pattern that explained most of the variance in men (7%)

We retained six factors, which were similar in both genders. Together, these factors explained 30% of the variance in the food intake data. The first derived factor explained 7.2% of the variance and was characterized by (i.e. loadings < − 0.25 or > 0.25) high intakes of fibre-rich bread, fruits, vegetables, breakfast cereals, fish and low-fat yoghurt, and by low intake of low-fibre bread, in both women and men. In men, the first factor was also characterized by high intake of cream, and in women by cottage cheese (Fig. 1a, b). In addition, rather low intakes of red and processed meat and sugar-sweetened beverages could be noted in both genders. This pattern was named the “health conscious” food pattern. The other five patterns (Supplementary Fig. 3a–j) were named the “low-fat products” pattern (characterized by high intakes of low-fat margarines, low-fat milk and low-fat yoghurt, but by low intake of butter, explained 5% of the variance), the “dressing and vegetables” pattern (characterized by high intake of dressing/oils, vegetables, poultry, salty snacks, rice/pasta, fried potatoes and cheese, but by low intake of boiled potatoes and jam/sugar, explained 5% of the variance), the “traditional meal” pattern (characterized by high intakes of egg, high-fat margarine, boiled potatoes, fish, red/processed meat, cream and high-fat milk, explained 5% of the variance in women and 4% in men), the “tea-breakfast” pattern (characterized by high intakes of tea and breakfast foods such as cereals, jam/sugar, high-fat yoghurt and high-fat milk, but by low intake of coffee and red/processed meat, explained 4% of the variance in women and 5% in men) and the “sugar-rich” pattern (characterized by high intakes of sweets, pastry, ice cream and sugar-sweetened beverages, explained 4% of the variance on both genders).

### Baseline characteristics

Baseline characteristics in the whole study sample and in the subsample with data on weight-change are shown in Supplementary Table 1. Women and men in the higher quintiles of the first food pattern (“health conscious”), which explained most of the variation in the food intakes, had higher age and reported higher intakes of energy, protein, carbohydrates, fibre and vitamin C, but lower intakes of fat and sucrose compared to individuals in the lower quintiles (Table 1). They were also characterized by rather favourable lifestyle characteristics including higher levels of leisure time physical activity and education, and a lower prevalence of smokers compared to those in the lower quintiles. Women adhering to this pattern had also higher BMI. In addition, some gender differences were observed regarding absolute intakes of foods in the lowest and highest quintile of the “health conscious” food pattern (Supplementary Table 2). Individuals adhering to the “low-fat products” pattern reported lower intakes of energy and fat, but higher BMI. Fewer among them had high education and they tended to smoke less and drink less alcohol. Those adhering to the “dressing and vegetables” pattern were younger, had higher BMI, diets with more polyunsaturated fat and protein, but less sugar. They tended to have higher education, smoke more and drink more alcohol. The other observed food patterns were also associated with baseline characteristics, although in a less consistent manner resulting in a less clear picture with regard to overall lifestyle (Supplementary Table 3).

**Table 1:** Baseline characteristics across quintiles (Q) of food patterns in men and women from the Malmö Diet and Cancer cohort

		Women (*n* = 12,456) Q of health conscious food pattern							Men (*n* = 8031) Q of HEALTH CONSCIOUS food pattern					
Baseline characteristics	Beta	1	2	3	4	5	*P* trend^a^	Beta	1	2	3	4	5	*P* trend^a^
Age (y)	+ 0.32	56.3	56.9	57.3	57.6	57.6	< 0.001	+ 0.33	57.9	58.8	58.8	59.1	59.4	< 0.001
BMI (kg/m^2)^	− 0.07	25.2	25.3	25.2	25.1	25.0	0.01	+ 0.05	26.0	26.1	26.0	26.1	26.2	0.09
Energy (MJ/day)	+ 0.23	8.1	8.4	8.7	8.9	9.1	< 0.001	+ 0.21	10.7	11.2	11.4	11.6	11.6	< 0.001
Protein (E%)	+ 0.30	14.9	15.2	14.4	15.6	16.2	< 0.001	+ 0.26	14.6	14.8	14.9	15.2	15.7	< 0.001
Fat (E%)	− 1.0	40.2	39.2	38.8	37.7	36.1	< 0.001	− 0.62	40.5	40.7	40.1	39.4	38.0	< 0.001
PUFA (E%)	− 0.14	6.2	6.0	6.0	5.8	5.6	< 0.001	− 0.10	6.4	6.5	6.3	6.2	6.0	< 0.001
Carbohydrate (E%)	+ 0.66	44.9	45.6	45.8	46.6	47.7	< 0.001	+ 0.36	44.9	44.5	45.0	45.4	46.2	< 0.001
Fibre (g/MJ)	+ 0.21	1.8	2.1	2.2	2.4	2.7	< 0.001	+ 0.14	1.6	1.8	1.9	2.0	2.2	< 0.001
Sucrose (E%)	− 0.16	9.1	8.9	8.5	8.6	8.4	< 0.001	− 0.25	8.6	8.2	8.2	7.8	7.6	< 0.001
Vitamin C (g/MJ)	+ 2.4	18	20	21	24	28	< 0.001	+ 1.6	10.9	11.2	13.3	15.7	16.7	< 0.001

							*P* value^b^							*P* value^b^
Alcohol intake, high (g/day)		2.7	2.7	2.6	2.2	2.1	0.39		7.7	8.3	7.7	7.7	6.9	0.71
Smokers, ex/current (%)		63.1	56.6	54.0	51.1	52.0	< 0.001		78.2	72.5	70.6	68.7	63.4	< 0.001
LTP^c^ activity, high (%)		13.8	17.5	19.4	20.8	25.2	< 0.001		15.1	18.2	18.2	21.4	24.6	< 0.001
Education, high (> 10 years) (%)		21.5	27.0	29.7	34.1	38.4	< 0.001		27.1	29.2	33.5	38.1	44.3	< 0.001

	Beta	Q of low-fat products pattern						Beta	Q of low-fat products pattern					
Age (y)	− 0.004	57.4	56.7	57.1	57.1	57.2	0.94	− 0.02	59.0	58.5	58.9	59.1	58.6	0.77
BMI (kg/m^2)^	+ 0.40	24.2	25.0	25.2	25.5	25.9	< 0.001	+ 0.21	25.5	26.0	26.1	26.3	26.4	< 0.001
Energy (MJ/day)	− 0.11	9.0	8.7	8.5	8.3	8.7	< 0.001	− 0.12	11.8	11.3	11.0	10.8	11.5	< 0.001
Protein (E%)	+ 0.41	14.4	15.3	15.6	15.8	16.2	< 0.001	+ 0.41	14.0	14.8	15.2	15.3	15.8	< 0.001
Fat (E%)	− 1.60	42.3	39.4	37.8	37.1	35.4	< 0.001	− 1.71	43.8	41.0	39.1	38.3	36.6	< 0.001
PUFA (E%)	+ 0.18	5.4	5.7	6.2	6.2	6.1	< 0.001	+ 0.27	5.5	6.0	6.7	6.7	6.5	< 0.001
Carbohydrate (E%)	+ 1.2	43.3	45.3	46.6	47.1	48.3	< 0.001	+ 1.3	42.2	44.2	45.7	46.3	47.6	< 0.001
Fibre (g/MJ)	+ 0.07	2.0	2.1	2.2	2.2	2.3	< 0.001	+ 0.08	1.7	1.8	2.0	2.0	2.0	< 0.001
Sucrose (E%)	+ 0.05	8.6	8.7	8.7	8.8	8.8	0.02	− 0.03	8.2	7.9	8.0	8.3	7.9	0.25
Vitamin C (g/MJ)	− 0.14	22.0	22.5	23.1	22.5	21.1	0.49	+ 0.02	13.2	13.2	14.7	14.1	12.9	0.91

							*P* value^b^							*P* value^b^
Alcohol intake, high (g/day)		2.8	3.5	2.6	2.0	1.4	< 0.001		11.1	9.7	7.0	5.8	4.8	< 0.001
Smokers, ex/current (%)		61.1	55.4	53.9	54.3	52.2	< 0.001		75.8	72.0	67.6	69.7	68.4	< 0.001
LTP^c^ activity, high (%)		20.2	20.4	19.1	18.1	19.0	0.25		17.4	19.1	20.6	21.4	19.1	0.04
Education, high (> 10 years) (%)		32.8	33.4	31.7	27.9	24.7	< 0.001		35.5	38.3	38.0	31.4	29.4	< 0.001

	Beta	Q of dressing/vegetables pattern						Beta	Q of dressing/vegetables pattern					
Age (years)	− 2.1	61.3	59.1	57.2	55.5	52.6	< 0.001	− 1.5	61.7	60.2	59.0	57.4	55.8	< 0.001
BMI (kg/m^2)^	+ 0.10	25.0	25.1	25.1	25.1	25.5	0.001	+ 0.12	25.8	26.0	25.9	26.2	26.3	< 0.001
Energy (MJ/day)	+ 0.03	8.5	8.6	8.7	8.7	8.6	0.06	− 0.06	11.4	11.3	11.4	11.3	11.1	0.01
Protein (E%)	+ 0.20	15.2	15.2	15.4	15.6	16.0	< 0.001	+ 0.26	14.5	14.8	15.1	15.2	15.6	< 0.001
Fat (E%)	+ 0.47	37.4	37.9	38.5	39.1	39.1	< 0.001	+ 0.35	38.9	39.5	39.9	40.2	40.3	< 0.001
PUFA (E%)	+ 0.29	5.2	5.7	6.0	6.2	6.5	< 0.001	+ 0.30	5.6	6.0	6.4	6.5	6.9	< 0.001
Carbohydrate (E%)	− 0.66	47.4	46.8	46.1	45.3	44.9	< 0.001	− 0.61	46.6	45.7	45.0	44.6	44.1	< 0.001
Fibre (g/MJ)	+ 0.02	2.1	2.2	2.2	2.2	2.3	< 0.001	+ 0.03	1.8	1.9	1.9	1.9	2.0	< 0.001
Sucrose (E%)	− 0.31	9.2	9.0	8.8	8.4	8.0	< 0.001	− 0.40	8.9	8.4	8.0	7.9	7.2	< 0.001
Vitamin C (g/MJ)	+ 1.3	18.9	21.0	23.2	24.1	23.8	< 0.001	+ 1.2	11.0	12.8	13.7	14.4	16.1	< 0.001

							*P* value^b^							*P* value^b^
Alcohol intake, high (g/day)		0.8	1.3	2.0	3.2	5.0	< 0.001		3.5	4.8	6.9	9.5	13.6	< 0.001
Smokers, ex/current (%)		49.5	51.2	56.1	57.4	62.6	< 0.001		71.4	71.7	70.0	69.4	71.0	0.58
LTP^c^ activity, high (%)		20.1	18.1	20.1	19.5	18.9	0.34		20.0	19.7	19.5	20.2	18.1	0.57
Education, high (> 10 years) (%)		15.6	23.8	28.1	37.2	45.9	< 0.001		19.4	27.9	34.4	43.0	47.6	< 0.001

^a^Calculated with the general linear model. Adjusted for age (continuous) when appropriate

^b^Chi-square test

^c^Leisure time physical activity, high = 5th quintile

### Food patterns and incidence of type 2 diabetes, coronary events, stroke and weight change

The “health conscious”, “low-fat products” and “dressing and vegetables” patterns showed significant associations with cardiometabolic diseases and the results regarding those patterns are presented in the main tables, whereas results regarding the other three patterns are presented as supplementary data.

#### Type 2 diabetes

In both genders, the “health conscious” pattern was associated with a significantly decreased incidence of T2D (HR comparing the highest quintile with the lowest: 0.75; 95% CI 0.61–0.92; *P* for trend across quintiles = 0.003 and HR 0.82; 95% CI 0.68–1.00; *P* for trend = 0.01 in women and men, respectively, full multivariable model) (Table 2). Concerning the “low-fat products” pattern, no significant associations were observed with incidence of T2D in the gender-specific analysis. However, as the “low-fat products” pattern tended to be associated with a somewhat higher risk in both genders, we also performed gender-combined analysis and observed that adherence to the “low-fat products” pattern was associated with increased risk of T2D (*P* for trend = 0.03). The other patterns did not show significant associations with T2D, but we observed a tendency of inverse association between the “tea-breakfast” pattern and T2D (*P* for trend = 0.07) in men, and a tendency of inverse association between the “sugar-rich” pattern and T2D in women (*P* for trend = 0.06) (Supplementary Table 4).

**Table 2:** Hazard ratios of type 2 diabetes across quintiles of dietary patterns in 12,456 women and 8031 men from the Malmö Diet and Cancer cohort

Quintiles of dietary patterns	Women		Men	
	Cases/person years	HR with 95% CIs	Cases/person years	HR with 95% CIs
Health conscious				
1	228/36,371	1.00	240/22,107	1.00
2	252/37,305	1.09 (0.91, 1.31)	240/22,879	0.98 (0.81, 1.17)
3	207/37,738	0.92 (0.76, 1.11)	234/23,070	0.95 (0.79–1.15)
4	216/38,289	0.96 (0.79, 1.16)	208/23,404	0.83 (0.68-1.00)
5	167/37,756	0.75 (0.61–0.92)	214/23,931	0.82 (0.68-1.00)
*P* trend across quintiles^a^		0.003		0.01
*P* trend, continuous score^ab^		0.003		0.01
Low-fat products				
1	173/37,574	1.00	196/22,663	1.00
2	198/37,726	1.03 (0.84, 1.26)	225/22,876	1.07 (0.88, 1.30)
3	218/38,000	0.75 (0.61, 0.92)	213/23,584	0.98 (0.80, 1.19)
4	208/37,648	1.02 (0.84, 1.26)	235/22,898	1.10 (0.90, 1.33)
5	273/37,845	1.19 (0.98, 1.45)	267/23,368	1.15 (0.96, 1.40)
*P* trend across quintiles^a^		0.10		0.12
*P* trend, continuous score^ab^		0.24		0.10
Dressing and vegetables				
1	247/36,617	1.00	214/21,924	1.00
2	229/37,666	1.02 (0.85, 1.22)	211/22,826	0.92 (0.76, 1.12)
3	199/38,219	0.92 (0.76, 1.11)	228/23,073	1.06 (0.87, 1.28)
4	187/38,149	0.96 (0.79, 1.18)	232/23,622	1.00 (0.82, 1.21)
5	208/38,144	1.12 (0.91, 1.37)	251/23,947	1.09 (0.90, 1.33)
*P* trend across quintiles^a^		0.54		0.25
*P* trend, continuous score^ab^		0.33		0.24

^a^Adjusted for age, season, diet method version, total energy intake, leisure time physical activity, smoking, alcohol intake, education, and baseline BMI

^b^*P* trend per unit of the pattern factor

#### Coronary events

The “health conscious” pattern was also inversely associated with incidence of coronary events (HR comparing the highest quintile with the lowest: 0.77; 95% CI 0.58–1.02; *P* for trend = 0.05 and HR 0.83; 95% CI 0.68–1.01; *P* for trend across quintiles = 0.07 in women and men, respectively, *P* for trend in gender-combined analysis = 0.006, full multivariable model) (Table 3). There seemed to be a threshold effect in men, indicating that it mainly were those with very low adherence to the health conscious food pattern who were at higher risk of coronary events. We observed an inverse association between the “dressing and vegetables” pattern and risk of coronary events (*P* for trend = 0.02) in men, but no similar tendency in women. In contrast, tendency of inverse association between the “sugar-rich” pattern and coronary events (*P* for trend = 0.053) was restricted to women (Supplementary Table 5).

**Table 3:** Hazard ratios of coronary events across quintiles of data driven dietary patterns in 12,456 women and 8031 men from the Malmö Diet and Cancer cohort

Quintiles of dietary patterns	Women		Men	
	Cases/person years	HR with 95% CIs	Cases/person years	HR with 95% CIs
Health conscious				
1	141/37,355	1.00	229/22,762	1.00
2	121/38,277	0.90 (0.70, 1.16)	198/23,642	0.82 (0.68, 1.00)
3	96/38,843	0.72 (0.54, 0.94)	190/23,964	0.79 (0.65, 0.96)
4	108/39,233	0.83 (0.64, 1.09)	191/24,021	0.80 (0.66, 0.98)
5	101/39,789	0.77 (0.58, 1.02)	196/24,365	0.83 (0.68, 1.01)
*P* trend across quintiles^a^		0.054		0.07
*P* trend, continuous score^ab^		0.03		0.02
Low-fat products				
1	111/38,107	1.00	191/23,285	1.00
2	97/38,633	0.91 (0.69, 1.19)	198/23,653	1.11 (0.91, 1.35)
3	119/39,065	1.11 (0.86, 1.44	180/24,496	1.00 (0.81, 1.22)
4	107/38,610	0.98 (0.75, 1.28	236/23,334	1.32 (1.09, 1.61)
5	133/39,084	1.18 (0.91, 1.52)))	199/24,288	1.06 (0.87, 1.30
*P* trend across quintiles^a^		0.17		0.19
*P* trend, continuous score^ab^		0.10		0.12
Dressing and vegetables				
1	165/37,711	1.00	249/22,319	1.00
2	140/38,473	1.07 (0.85, 1.34)	247/23,253	1.07 (0.90, 1.28)
3	104/39,144	0.92 (0.72, 1.18)	193/23.762	0.91 (0.75, 1.10)
4	82/39,019	0.92 (0.69, 1.21)	172/24,495	0.87 (0.71, 1.07)
5	76/39,151	1.13 (0.84, 1.53)	143/24,925	0.83 (0.66, 1.03)
*P* trend across quintiles^a^		0.99		0.02
*P* trend, continuous score^ab^		0.66		0.01

^a^Adjusted for age, season, diet method version, total energy intake, leisure time physical activity, smoking, alcohol intake, education, and baseline BMI

^b^*P* trend per unit of the pattern factor

#### Stroke

The “health conscious” pattern was associated with decreased incidence of stroke (HR comparing the highest quintile with the lowest: 0.69; 95% CI 0.54–0.88; *P* for trend across quintiles = 0.001, full multivariable model) (Table 4) in men, but no such tendencies were seen in women. None of the other obtained food patterns was associated with stroke (Table 4, Supplementary Table 6).

**Table 4:** Hazard ratios of stroke across quintiles of data driven dietary patterns in 12,456 women and 8031 men from the Malmö Diet and Cancer cohort

Quintiles of dietary patterns	Women		Men	
	Cases/person years	HR with 95% CIs	Cases/person years	HR with 95% CIs
Health conscious				
1	138/37,128	1.00	156/23,009	1.00
2	145/38,187	1.02 (0.81, 1.30)	142/23,807	0.83 (0.66, 1.04)
3	130/38,539	0.92 (0.72, 1.17)	125/24,032	0.72 (0.56, 0.91)
4	121/39,111	0.88 (0.68, 1.27)	123/24,247	0.70 (0.55, 0.89)
5	131/39,594	0.96 (0.75, 1.24)	121/24,647	0.69 (0.54, 0.88)
*P* trend across quintiles^a^		0.42		0.001
*P* trend, continuous score^ab^		0.63		0.001
Low-fat products				
1	146/37,925	1.00	145/23,329	1.00
2	132/38,359	0.94 (0.74, 1.19)	138/23,733	1.02 (0.80, 1.29)
3	128/38,853	0.88 (0.70, 1.12)	125/24,352	0.89 (0.70, 1.14)
4	130/38,361	0.91 (0.72, 1.16)	136/23,725	0.99 (0.78, 1.26)
5	129/39,056	0.87 (0.68, 1.11)	123/24,602	0.87 (0.68, 1.11)
*P* trend across quintiles^a^		0.26		0.26
*P* trend, continuous score^ab^		0.20		0.37
Dressing and vegetables				
1	192/37,412	1.00	168/22,622	1.00
2	165/38,242	1.03 (0.74, 1.27)	143/23,497	0.96 (0.77, 1.21)
3	130/38,910	0.96 (0.76, 1.20)	136/23,880	0.98 (0.78, 1.24)
4	100/38,929	0.88 (0.68, 1.14)	121/24,633	0.97 (0.76, 1.25)
5	78/39,066	0.95 (0.71, 1.27)	99/25,109	0.91 (0.70, 1.19)
*P* trend across quintiles^a^		0.38		0.59
*P* trend, continuous score^ab^		0.62		0.33

^a^Adjusted for age, season, diet method version, total energy intake, leisure time physical activity, smoking, alcohol intake, education, and baseline BMI

^b^*P* trend per unit of the pattern factor

#### Weight change

The women gained on average 2.5 kg during follow-up, whereas men gained 1.7 kg.

In men, the “health conscious” food pattern was associated with less pronounced weight gain during the 17-year follow-up (0.93 kg less/10 years in the highest compared with the lowest quintile; *P* for trend across the quintiles = 0.03) (Table 5). The “tea-breakfast” pattern tended to associate with less pronounced weight gain in women (Supplementary Table 7).

**Table 5:** Weight change during follow-up in quintiles of data driven dietary patterns in women and men from the Malmö Diet and Cancer cohort

	10-year weight change^a^	
Quintiles of dietary patterns	Women (*n* = 1533)	Men (*n* = 1094)
Health conscious		
1	1.93^a^ ± 1.26	2.04^a^ ± 1.17
2	2.10^a^ ± 1.27	1.65^ab^ ± 1.17
3	2.30^a^ ± 1.25	1.12^b^ ± 1.17
4	1.96^a^ ± 1.26	1.40^b^ ± 1.16
5	1.97^a^ ± 1.25	1.11^b^ ± 1.15
*P* trend across quintiles	0.81	0.03
*P* trend, continuous score^b^	0.89	0.09
Low-fat products		
1	1.77^a^ ± 1.25	1.11^a^ ± 1.18
2	2.45^a^ ± 1.25	1.44^a^ ± 1.16
3	2.07^a^ ± 1.26	1.42^a^ ± 1.16
4	2.12^a^ ± 1.26	1.14^a^ ± 1.17
5	2.15^a^ ± 1.25	1.14^a^ ± 1.17
*P* trend across quintiles	0.68	0.76
*P* trend, continuous score^b^	0.91	0.78
Dressing and vegetables		
1	1.96^a^ ± 1.25	1.31^a^ ± 1.18
2	1.84^a^ ± 1.26	1.56^a^ ± 1.17
3	1.81^a^ ± 1.25	1.28^a^ ± 1.16
4	1.90^a^ ± 1.26	1.08^a^ ± 1.16
5	2.46^b^ ± 1.25	1.55^a^ ±1.16
*P* trend across quintiles	0.20	0.99
*P* trend, continuous score^b^	0.16	0.93

^a^Homogenous subsets are indicated by letters. Adjusted for age, season, diet method version, total energy intake, leisure time physical activity, smoking, alcohol intake, education, and baseline BMI

^b^*P* trend per unit of the pattern factor

Neither exclusion of BMI from the full multivariable model, nor inclusion of baseline weight or waist circumference had any major influence on any of the results. Finally, we did not observe any significant interactions with gender or BMI status.

#### Sensitivity analyses

After exclusion of individuals above 60 years of age at baseline (38% of the women and 43% of the men), the “health conscious” pattern remained significantly associated with lower risk of T2D in both women and men, and with lower risk of stroke in men. Regarding the “health conscious” pattern and coronary events, the risk estimates were found to be similar to those in the main analysis although non-significant. However, to gain power we also performed gender combined analysis and then observed a clear tendency of inverse association (HR comparing the highest quintile with the lowest: 0.79; 95% CI 0.61, 1.02; *P* trend = 0.07).

The “low-fat-products” pattern was no longer associated with higher risk of T2D after excluding those above 60 years of age (HR comparing the highest quintile with the lowest: 1.02; 95% CI 0.86, 1.22; *P* trend = 0.53 in gender-combined analysis).

In men, adherence to the “dressing and vegetable” pattern was in men more strongly associated with lower risk of coronary events after excluding those above 60 years of age (HR comparing the highest quintile with the lowest: 0.63; 95% CI 0.45, 0.80; *P* trend = 0.01).

## Discussion

In this large study, using principal component analysis to derive food patterns from the Malmö Diet and Cancer cohort, we observed similar patterns in both women and men, suggesting that the patterns are fairly robust. A dietary pattern characterized by health-conscious food choices, such as plant foods, fish and low-fat yoghurt, was associated with decreased incidence of T2D and coronary events in both genders. In men, the “health conscious” food pattern was also associated with decreased risk of stroke and with less pronounced weight gain during 17-year follow-up. A pattern mainly characterized by dressing and vegetables was associated with lower risk of coronary events in men. The other retained food patterns did not show any significant associations with cardiometabolic diseases in women or men, although adherence to the “low-fat products” pattern was associated with higher risk of T2D in gender-combined analysis.

In line with our observation, previous studies have also observed data-driven healthy dietary patterns, mainly characterized by high intakes of plant foods such as fruits, vegetables and whole grain, to associate with lower incidence of both T2D [4] and coronary events [5]. Nevertheless, a need for additional studies to confirm the findings has been declared and associations with stroke have been less consistent [5]. The differing observations regarding stroke may be explained by dissimilarities in foods characterizing the healthy patterns; healthy/prudent food patterns have indeed indicated protective associations with stroke in several Western populations [24–26], whereas the findings in Asian populations are less convincing [27–29]. Retained healthy food patterns in Asia are in line with other healthy food patterns defined by high intakes of various fruits and vegetables, but in contrast to a lesser degree by whole grain foods. It is thereby possible that the value of capturing intake of several healthy foods, with various health-promoting properties, will partly be missed. In contrast to previous reports from Western cohorts, our finding of an inverse association between the “health conscious” food pattern and decreased risk of stroke was restricted to men and no such tendencies were seen in women. Gender differences in intake levels of various foods could at least partially explain the differing observations. Intakes of fruits and vegetables in the MDC cohort seem, for example, to be higher in women than in men [30], whereas intake of refined bread seem to be higher in men [31], and it is possible that intake of healthy foods may lie above potential critical threshold levels with regard to stroke in most women. Absolute intake ranges of healthy foods between the lowest and highest quintile of the “health conscious” food pattern may also be of importance; in men, mean estimated daily intake of fibre-rich bread in the quintiles ranged, for example, from 7 to 70 g, whereas it ranged from 10 to 60 g in women. In agreement with our observations of that men with highest adherence to the health conscious food pattern gained almost 1 kg less weight per 10 years compared to men with lowest adherence, a dietary pattern loading high in fruits and vegetables, whole grain products and fish associated with less weight gain in Australian men, but not women [6]. Furthermore, no overall association was seen between a vegetable/fruit pattern and weight-change in African-American women [32]. On the other hand, in other studies, patterns similar to our “health conscious” pattern have been associated with smaller weight gain also in women [8, 9, 33]. Biological mechanisms underlying our observed associations between the “health conscious” pattern and cardiometabolic disease may include effects on satiety, glucose and lipid metabolism, and oxidative stress, due to higher intake of plant food components such as fibre and phytochemicals, but lower sugar intake [34–36]. Effects on gut microbiota by fibre and yoghurt may also play a role [37, 38].

Consistent with our findings regarding the “health conscious” food pattern, an a priori-defined high diet quality index, based on Swedish nutrition recommendations (SNR-DQI), is associated with lower incidence of cardiovascular disease in the MDC cohort [39]. Nevertheless, the SNR-DQI did not associate with risk of T2D [40]. Similar to characteristics of our data-driven “health conscious” food pattern, the SNR-DQI was defined by intake levels of fibre, fruits, vegetables and fish, but in contrast also by intakes of sucrose and different types of fat, which may explain the differing associations. The evidence regarding the importance of fat quality is indeed more convincing with regard to the risk of cardiovascular disease compared to that of T2D [16]. In this study, the “dressing and vegetables” pattern, which includes oil-based dressings, was characterized by a markedly higher dietary content of polyunsaturated fat. Men adhering to this pattern had indeed a lower risk for coronary events. In contrast, the “low-fat products” food pattern tended to associate with higher risk of T2D. However, we cannot exclude that these findings could be a result of reverse causation and the association did not persist when excluding individuals above 60 years of age. It is possible that many of the individuals adhering to this pattern choose low-fat products to lose weight and dietary habits earlier in life may explain the high BMI as well as their higher risk of T2D, because BMI at baseline was actually higher among those adhering to this pattern. On the other hand, the findings regarding the “low-fat product” pattern are in line with those of a previous findings, suggesting that some dairy fats may contribute to lowering the risk of T2D [41, 42]. Risk factors may indeed differ between T2D and coronary disease and there is, for example, strong evidence for that the role of LDL cholesterol differs, as Mendelian randomization studies indicate that low LDL cholesterol is associated with hyperglycaemia and increased risk of T2D [43].

Our study has several strengths. It is a large study with long follow-up time. Moreover, it is a population-based prospective study, which reduces the risk of selection bias and reverse causation (although as discussed above it cannot be ruled out). The relative validity of food intakes of importance in this study indicates dietary data of high quality [13, 14]. Further strengths are the extensive information on potential confounders, and that diet was measured with a modified diet history method including a 7-day food record for cooked meals. In other studies reporting associations between data-driven dietary patterns and cardiometabolic disease, diet has almost exclusively been measured using food frequency questionnaires, and it may be valuable to also report findings from studies using other assessment methods, as the method used may affect obtained food patterns, especially if predefined food lists are used. Different measurement methods are also associated with differing measurement errors that could affect the results. Finally, we observed similar food patterns in women and men, indicating robust patterns.

A limitation of the study is that diet and other lifestyle factors were only measured at baseline. Another drawback may be potentially subjective decisions regarding the grouping of food variables to include in the principal component analysis, as it could influence the obtained dietary patterns. Our aim was to cover as many parts of the overall diet as possible, but to have a less detailed level on foods reported during the record period and known to be consumed irregularly, as they may be unsatisfactorily captured on a 7-day basis if they are not aggregated. This was, for example, the reason to why we grouped fatty and lean fish together. Although it has been indicated that the number of input variables do not have any major effect on the derived food patterns [44], we cannot exclude that a different detail level regarding the food classification would have revealed somewhat altered patterns. Another concern is that dietary patterns could represent overall lifestyle, and despite adjustment for several confounders, we cannot completely rule out residual confounding. Finally, findings from dietary pattern analyses may be explained by specific foods or nutrients related to the pattern, and it can be challenging to reveal whether certain components lie behind the results or whether the whole pattern is crucial [45].

The “health conscious” pattern, which explained most of the variance in food intakes in this study, was strongest related to several foods that per se have been found to associate with cardiometabolic disease in other studies [34–36, 46–50]. Furthermore, these foods have previously been included in predefined healthy diet indexes, suggesting that the “health conscious” pattern may also be useful when examining associations with other chronic disease such as different types of cancer. Future studies will reveal the relevance of our observations indicating that patterns characterized low-fat products and fat quality may be differently related to T2D compared to coronary disease.

To conclude, our findings indicate that adhering to a “health conscious” food pattern, characterized by high intake of fibre-rich plant foods, fish and low-fat yoghurt, but low intake of low-fibre bread, sugar-sweetened beverages, and red and processed meat, which is in line with European dietary guidelines [51], may contribute to decreased risk of cardiometabolic disease and especially to decreased risk of T2D.

## Electronic supplementary material

Below is the link to the electronic supplementary material.

Supplementary material 1 (DOCX 35 KB)

Supplementary material 2 (DOCX 43 KB)

Supplementary material 3 (DOCX 327 KB)

Supplementary material 4 (DOCX 43 KB)
