# Supplementary material for: Food patterns in relation to weight change and incidence of type 2 diabetes, coronary events and stroke in the Malmö Diet and Cancer cohort
Source: Eur J Nutr. 2018 May 31;58(5):1801–14. doi: 10.1007/s00394-018-1727-9 (PMC6647222; doi:10.1007/s00394-018-1727-9)
Supplement: Supplementary file 1 — Supplementary material 1 (DOCX 35 KB) [file 394_2018_1727_MOESM1_ESM.docx]

Supplementary figure 1.
