# Supplementary material for: Food patterns in relation to weight change and incidence of type 2 diabetes, coronary events and stroke in the Malmö Diet and Cancer cohort
Source: Eur J Nutr. 2018 May 31;58(5):1801–14. doi: 10.1007/s00394-018-1727-9 (PMC6647222; doi:10.1007/s00394-018-1727-9)

Supplementary figure 2a. Scree plot from principal component analysis in 12,463 women from the Malmö Diet and Cancer study (eigenvalues >1). Reduction of 33 energy-adjusted food groups into factors representing food patterns.
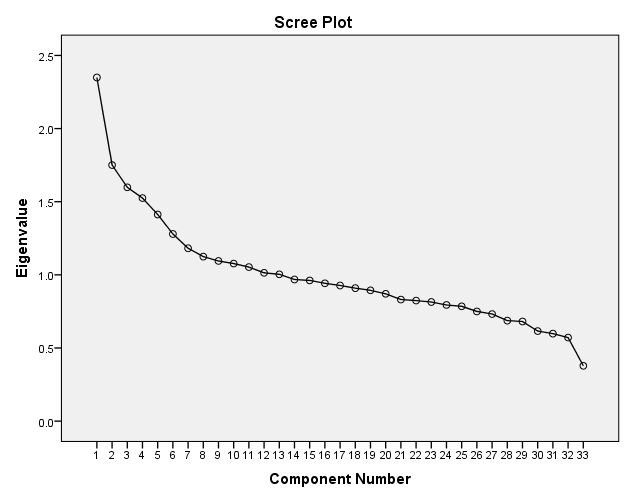


Supplementary figure 2b. Scree plot from principal component analysis in 8,037 men from the Malmö Diet and Cancer study (eigenvalues >1). Reduction of 33 energy-adjusted food groups into factors representing food patterns.
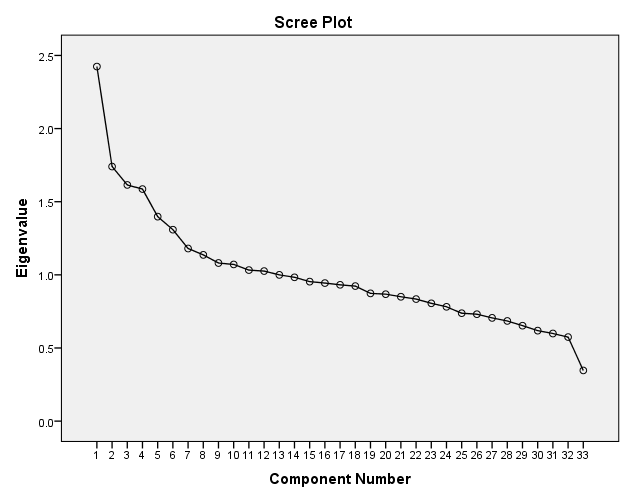

Supplement: Supplementary file 2 — Supplementary material 2 (DOCX 43 KB) [file 394_2018_1727_MOESM2_ESM.docx]
