# Supplementary material for: Food patterns in relation to weight change and incidence of type 2 diabetes, coronary events and stroke in the Malmö Diet and Cancer cohort
Source: Eur J Nutr. 2018 May 31;58(5):1801–14. doi: 10.1007/s00394-018-1727-9 (PMC6647222; doi:10.1007/s00394-018-1727-9)
Supplement: Supplementary file 3 — Supplementary material 3 (DOCX 327 KB) [file 394_2018_1727_MOESM3_ESM.docx]

Supplementary Figure 3a.

Supplementary Figure 3b.

Supplementary Figure 3c.

Supplementary Figure 3d.

Supplementary Figure 3e.

Supplementary Figure 3f.****

Supplementary Figure 3g.

Supplementary Figure 3h.

Supplementary Figure 3i.

Supplementary Figure 3j.
