# Supplementary material for: Food patterns in relation to weight change and incidence of type 2 diabetes, coronary events and stroke in the Malmö Diet and Cancer cohort
Source: Eur J Nutr. 2018 May 31;58(5):1801–14. doi: 10.1007/s00394-018-1727-9 (PMC6647222; doi:10.1007/s00394-018-1727-9)
Supplement: Supplementary file 4 — Supplementary material 4 (DOCX 43 KB) [file 394_2018_1727_MOESM4_ESM.docx]

**Table 1.** Baseline characteristics across quintiles (Q) of food patterns in men and women from the Malmö Diet and Cancer cohort

|  |  | Women (n=12,456)  Q of Health conscious food pattern | | | | | |  | Men (n=8,031)  Q of Health conscious food pattern | | | | | |
| --- | --- | --- | --- | --- | --- | --- | --- | --- | --- | --- | --- | --- | --- | --- |
| Baseline characteristics | Beta | 1 | 2 | 3 | 4 | 5 | P-trend^a^ | Beta | 1 | 2 | 3 | 4 | 5 | P-trend^a^ |
| Age (y) | +0.32 | 56.3 | 56.9 | 57.3 | 57.6 | 57.6 | <0.001 | +0.33 | 57.9 | 58.8 | 58.8 | 59.1 | 59.4 | <0.001 |
| BMI (kg/m^2)^ | -0.07 | 25.2 | 25.3 | 25.2 | 25.1 | 25.0 | 0.01 | +0.05 | 26.0 | 26.1 | 26.0 | 26.1 | 26.2 | 0.09 |
| Energy (MJ/d) | +0.23 | 8.1 | 8.4 | 8.7 | 8.9 | 9.1 | <0.001 | +0.21 | 10.7 | 11.2 | 11.4 | 11.6 | 11.6 | <0.001 |
| Protein (E%) | +0.30 | 14.9 | 15.2 | 14.4 | 15.6 | 16.2 | <0.001 | +0.26 | 14.6 | 14.8 | 14.9 | 15.2 | 15.7 | <0.001 |
| Fat (E%) | -1.0 | 40.2 | 39.2 | 38.8 | 37.7 | 36.1 | <0.001 | -0.62 | 40.5 | 40.7 | 40.1 | 39.4 | 38.0 | <0.001 |
| PUFA (E%) | -0.14 | 6.2 | 6.0 | 6.0 | 5.8 | 5.6 | <0.001 | -0.10 | 6.4 | 6.5 | 6.3 | 6.2 | 6.0 | <0.001 |
| Carbohydrate (E%) | +0.66 | 44.9 | 45.6 | 45.8 | 46.6 | 47.7 | <0.001 | +0.36 | 44.9 | 44.5 | 45.0 | 45.4 | 46.2 | <0.001 |
| Fibre (g/MJ) | +0.21 | 1.8 | 2.1 | 2.2 | 2.4 | 2.7 | <0.001 | +0.14 | 1.6 | 1.8 | 1.9 | 2.0 | 2.2 | <0.001 |
| Sucrose (E%) | -0.16 | 9.1 | 8.9 | 8.5 | 8.6 | 8.4 | <0.001 | -0.25 | 8.6 | 8.2 | 8.2 | 7.8 | 7.6 | <0.001 |
| Vitamin C (g/MJ) | +2.4 | 18 | 20 | 21 | 24 | 28 | <0.001 | +1.6 | 10.9 | 11.2 | 13.3 | 15.7 | 16.7 | <0.001 |
|  |  |  |  |  |  |  | P-value^b^ |  |  |  |  |  |  | P-value^b^ |
| Alcohol intake, high (g/d) |  | 2.7 | 2.7 | 2.6 | 2.2 | 2.1 | 0.39 |  | 7.7 | 8.3 | 7.7 | 7.7 | 6.9 | 0.71 |
| Smokers, ex/current (%) |  | 63.1 | 56.6 | 54.0 | 51.1 | 52.0 | <0.001 |  | 78.2 | 72.5 | 70.6 | 68.7 | 63.4 | <0.001 |
| LTP^c^ activity, high (%) |  | 13.8 | 17.5 | 19.4 | 20.8 | 25.2 | <0.001 |  | 15.1 | 18.2 | 18.2 | 21.4 | 24.6 | <0.001 |
| Education, high (>10y) (%) |  | 21.5 | 27.0 | 29.7 | 34.1 | 38.4 | <0.001 |  | 27.1 | 29.2 | 33.5 | 38.1 | 44.3 | <0.001 |
|  |  |  |  |  |  |  |  |  |  |  |  |  |  |  |
|  | Beta | Q of Low-fat products pattern | | | | | | Beta | Q of Low-fat products pattern | | | | | |
| Age (y) | -0.004 | 57.4 | 56.7 | 57.1 | 57.1 | 57.2 | 0.94 | -0.02 | 59.0 | 58.5 | 58.9 | 59.1 | 58.6 | 0.77 |
| BMI (kg/m^2)^ | +0.40 | 24.2 | 25.0 | 25.2 | 25.5 | 25.9 | <0.001 | +0.21 | 25.5 | 26.0 | 26.1 | 26.3 | 26.4 | <0.001 |
| Energy (MJ/d) | -0.11 | 9.0 | 8.7 | 8.5 | 8.3 | 8.7 | <0.001 | -0.12 | 11.8 | 11.3 | 11.0 | 10.8 | 11.5 | <0.001 |
| Protein (E%) | +0.41 | 14.4 | 15.3 | 15.6 | 15.8 | 16.2 | <0.001 | +0.41 | 14.0 | 14.8 | 15.2 | 15.3 | 15.8 | <0.001 |
| Fat (E%) | -1.60 | 42.3 | 39.4 | 37.8 | 37.1 | 35.4 | <0.001 | -1.71 | 43.8 | 41.0 | 39.1 | 38.3 | 36.6 | <0.001 |
| PUFA (E%) | +0.18 | 5.4 | 5.7 | 6.2 | 6.2 | 6.1 | <0.001 | +0.27 | 5.5 | 6.0 | 6.7 | 6.7 | 6.5 | <0.001 |
| Carbohydrate (E%) | +1.2 | 43.3 | 45.3 | 46.6 | 47.1 | 48.3 | <0.001 | +1.3 | 42.2 | 44.2 | 45.7 | 46.3 | 47.6 | <0.001 |
| Fibre (g/MJ) | +0.07 | 2.0 | 2.1 | 2.2 | 2.2 | 2.3 | <0.001 | +0.08 | 1.7 | 1.8 | 2.0 | 2.0 | 2.0 | <0.001 |
| Sucrose (E%) | +0.05 | 8.6 | 8.7 | 8.7 | 8.8 | 8.8 | 0.02 | -0.03 | 8.2 | 7.9 | 8.0 | 8.3 | 7.9 | 0.25 |
| Vitamin C (g/MJ) | -0.14 | 22.0 | 22.5 | 23.1 | 22.5 | 21.1 | 0.49 | +0.02 | 13.2 | 13.2 | 14.7 | 14.1 | 12.9 | 0.91 |
|  |  |  |  |  |  |  |  |  |  |  |  |  |  |  |
|  |  |  |  |  |  |  | P-value^b^ |  |  |  |  |  |  | P-value^b^ |
| Alcohol intake, high (g/d) |  | 2.8 | 3.5 | 2.6 | 2.0 | 1.4 | <0.001 |  | 11.1 | 9.7 | 7.0 | 5.8 | 4.8 | <0.001 |
| Smokers, ex/current (%) |  | 61.1 | 55.4 | 53.9 | 54.3 | 52.2 | <0.001 |  | 75.8 | 72.0 | 67.6 | 69.7 | 68.4 | <0.001 |
| LTP^c^ activity, high (%) |  | 20.2 | 20.4 | 19.1 | 18.1 | 19.0 | 0.25 |  | 17.4 | 19.1 | 20.6 | 21.4 | 19.1 | 0.04 |
| Education, high (>10y) (%) |  | 32.8 | 33.4 | 31.7 | 27.9 | 24.7 | <0.001 |  | 35.5 | 38.3 | 38.0 | 31.4 | 29.4 | <0.001 |
|  |  |  |  |  |  |  |  |  |  |  |  |  |  |  |
|  | Beta | Q of Dressing/vegetables pattern | | | | | | Beta | Q of Dressing/vegetables pattern | | | | | |
| Age (y) | -2.1 | 61.3 | 59.1 | 57.2 | 55.5 | 52.6 | <0.001 | -1.5 | 61.7 | 60.2 | 59.0 | 57.4 | 55.8 | <0.001 |
| BMI (kg/m^2)^ | +0.10 | 25.0 | 25.1 | 25.1 | 25.1 | 25.5 | 0.001 | +0.12 | 25.8 | 26.0 | 25.9 | 26.2 | 26.3 | <0.001 |
| Energy (MJ/d) | +0.03 | 8.5 | 8.6 | 8.7 | 8.7 | 8.6 | 0.06 | -0.06 | 11.4 | 11.3 | 11.4 | 11.3 | 11.1 | 0.01 |
| Protein (E%) | +0.20 | 15.2 | 15.2 | 15.4 | 15.6 | 16.0 | <0.001 | +0.26 | 14.5 | 14.8 | 15.1 | 15.2 | 15.6 | <0.001 |
| Fat (E%) | +0.47 | 37.4 | 37.9 | 38.5 | 39.1 | 39.1 | <0.001 | +0.35 | 38.9 | 39.5 | 39.9 | 40.2 | 40.3 | <0.001 |
| PUFA (E%) | +0.29 | 5.2 | 5.7 | 6.0 | 6.2 | 6.5 | <0.001 | +0.30 | 5.6 | 6.0 | 6.4 | 6.5 | 6.9 | <0.001 |
| Carbohydrate (E%) | -0.66 | 47.4 | 46.8 | 46.1 | 45.3 | 44.9 | <0.001 | -0.61 | 46.6 | 45.7 | 45.0 | 44.6 | 44.1 | <0.001 |
| Fibre (g/MJ) | +0.02 | 2.1 | 2.2 | 2.2 | 2.2 | 2.3 | <0.001 | +0.03 | 1.8 | 1.9 | 1.9 | 1.9 | 2.0 | <0.001 |
| Sucrose (E%) | -0.31 | 9.2 | 9.0 | 8.8 | 8.4 | 8.0 | <0.001 | -0.40 | 8.9 | 8.4 | 8.0 | 7.9 | 7.2 | <0.001 |
| Vitamin C (g/MJ) | +1.3 | 18.9 | 21.0 | 23.2 | 24.1 | 23.8 | <0.001 | +1.2 | 11.0 | 12.8 | 13.7 | 14.4 | 16.1 | <0.001 |
|  |  |  |  |  |  |  |  |  |  |  |  |  |  |  |
|  |  |  |  |  |  |  | P-value^b^ |  |  |  |  |  |  | P-value^b^ |
| Alcohol intake, high (g/d) |  | 0.8 | 1.3 | 2.0 | 3.2 | 5.0 | <0.001 |  | 3.5 | 4.8 | 6.9 | 9.5 | 13.6 | <0.001 |
| Smokers, ex/current (%) |  | 49.5 | 51.2 | 56.1 | 57.4 | 62.6 | <0.001 |  | 71.4 | 71.7 | 70.0 | 69.4 | 71.0 | 0.58 |
| LTP^c^ activity, high (%) |  | 20.1 | 18.1 | 20.1 | 19.5 | 18.9 | 0.34 |  | 20.0 | 19.7 | 19.5 | 20.2 | 18.1 | 0.57 |
| Education, high (>10y) (%) |  | 15.6 | 23.8 | 28.1 | 37.2 | 45.9 | <0.001 |  | 19.4 | 27.9 | 34.4 | 43.0 | 47.6 | <0.001 |

^a^Calculated with the general linear model. Adjusted for age (continuous) when appropriate.

^b^Chi-square test.

^c^Leisure time physical activity, high=5^th^ quintile

Table 2. Hazard ratios of type 2 diabetes across quintiles of dietary patterns in 12,456 women and 8,031 men from the Malmö Diet and Cancer cohort.

| Quintiles of dietary patterns | women | | men | |
| --- | --- | --- | --- | --- |
|  | cases/  person ys | HR  with 95% CIs | cases/  person ys | HR  with 95% CIs |
| Health conscious |  |  |  |  |
| 1 | 228/36,371 | 1.00 | 240/22,107 | 1.00 |
| 2 | 252/37,305 | 1.09  (0.91, 1.31) | 240/22,879 | 0.98  (0.81, 1.17) |
| 3 | 207/37,738 | 0.92  (0.76, 1.11) | 234/23,070 | 0.95  (0.79-1.15) |
| 4 | 216/38,289 | 0.96  (0.79, 1.16) | 208/23,404 | 0.83  (0.68-1.00) |
| 5 | 167/37,756 | 0.75  (0.61-0.92) | 214/23,931 | 0.82  (0.68-1.00) |
| *P-trend across quintiles*^a^ |  | *0.003* |  | *0.01* |
| *P-trend, continuous score*^ab^ |  | *0.003* |  | *0.01* |
|  |  |  |  |  |
| Low-fat products |  |  |  |  |
| 1 | 173/37,574 | 1.00 | 196/22,663 | 1.00 |
| 2 | 198/37,726 | 1.03  (0.84, 1.26) | 225/22,876 | 1.07  (0.88, 1.30) |
| 3 | 218/38,000 | 0.75  (0.61, 0.92) | 213/23,584 | 0.98  (0.80, 1.19) |
| 4 | 208/37,648 | 1.02  (0.84, 1.26) | 235/22,898 | 1.10  (0.90, 1.33) |
| 5 | 273/37,845 | 1.19  (0.98, 1.45) | 267/23,368 | 1.15  (0.96, 1.40) |
| *P-trend across quintiles*^a^ |  | *0.10* |  | *0.12* |
| *P-trend, continuous score*^ab^ |  | *0.24* |  | *0.10* |
|  |  |  |  |  |
| Dressing and vegetables |  |  |  |  |
| 1 | 247/36,617 | 1.00 | 214/21,924 | 1.00 |
| 2 | 229/37,666 | 1.02  (0.85, 1.22) | 211/22,826 | 0.92  (0.76, 1.12) |
| 3 | 199/38,219 | 0.92  (0.76, 1.11) | 228/23,073 | 1.06  (0.87, 1.28) |
| 4 | 187/38,149 | 0.96  (0.79, 1.18) | 232/23,622 | 1.00  (0.82, 1.21) |
| 5 | 208/38,144 | 1.12  (0.91, 1.37) | 251/23,947 | 1.09  (0.90, 1.33) |
| *P-trend across quintiles*^a^ |  | *0.54* |  | *0.25* |
| *P-trend, continuous score*^ab^ |  | *0.33* |  | *0.24* |

^a^Adjusted for age, season, diet method version, total energy intake, leisure time physical activity, smoking, alcohol intake, education, and baseline BMI

^b^P-trend per unit of the pattern factor

Table 3. Hazard ratios of coronary events across quintiles of data driven dietary patterns in 12,456 women and 8,031 men from the Malmö Diet and Cancer cohort.

| Quintiles of dietary patterns | women | | men | |
| --- | --- | --- | --- | --- |
|  | cases/  person ys | HR  with 95% CIs | cases/  person ys | HR  with 95% CIs |
| Health conscious |  |  |  |  |
| 1 | 141/37,355 | 1.00 | 229/22,762 | 1.00 |
| 2 | 121/38,277 | 0.90  0.70, 1.16 | 198/23,642 | 0.82  0.68, 1.00 |
| 3 | 96/38,843 | 0.72  0.54, 0.94 | 190/23,964 | 0.79  0.65, 0.96 |
| 4 | 108/39,233 | 0.83  0.64, 1.09 | 191/24,021 | 0.80  0.66, 0.98 |
| 5 | 101/39,789 | 0.77  0.58, 1.02 | 196/24,365 | 0.83  0.68, 1.01 |
| *P-trend across quintiles*^a^ |  | *0.054* |  | *0.07* |
| *P-trend, continuous score*^ab^ |  | *0.03* |  | *0.02* |
|  |  |  |  |  |
| Low-fat products |  |  |  |  |
| 1 | 111/38,107 | 1.00 | 191/23,285 | 1.00 |
| 2 | 97/38,633 | 0.91  0.69, 1.19 | 198/23,653 | 1.11  0.91, 1.35 |
| 3 | 119/39,065 | 1.11  0.86, 1.44 | 180/24,496 | 1.00  0.81, 1.22 |
| 4 | 107/38,610 | 0.98  0.75, 1.28 | 236/23,334 | 1.32  1.09, 1.61 |
| 5 | 133/39,084 | 1.18  0.91, 1.52 | 199/24,288 | 1.06  0.87, 1.30 |
| *P-trend across quintiles*^a^ |  | *0.17* |  | *0.19* |
| *P-trend, continuous score*^ab^ |  | *0.10* |  | *0.12* |
|  |  |  |  |  |
| Dressing and vegetables |  |  |  |  |
| 1 | 165/37,711 | 1.00 | 249/22,319 | 1.00 |
| 2 | 140/38,473 | 1.07  0.85, 1.34 | 247/23,253 | 1.07  0.90, 1.28 |
| 3 | 104/39,144 | 0.92  0.72, 1.18 | 193/23.762 | 0.91  0.75, 1.10 |
| 4 | 82/39,019 | 0.92  0.69, 1.21 | 172/24,495 | 0.87  0.71, 1.07 |
| 5 | 76/39,151 | 1.13  0.84, 1.53 | 143/24,925 | 0.83  0.66, 1.03 |
| *P-trend across quintiles*^a^ |  | *0.99* |  | *0.02* |
| *P-trend, continuous score*^ab^ |  | *0.66* |  | *0.01* |

^a^Adjusted for age, season, diet method version, total energy intake, leisure time physical activity, smoking, alcohol intake, education, and baseline BMI

^b^P-trend per unit of the pattern factor

Table 4. Hazard ratios of stroke across quintiles of data driven dietary patterns in 12,456 women and 8,031 men from the Malmö Diet and Cancer cohort.

| Quintiles of dietary patterns | women | | men | |
| --- | --- | --- | --- | --- |
|  | cases/  person ys | HR  with 95% CIs | cases/  person ys | HR  with 95% CIs |
| Health conscious |  |  |  |  |
| 1 | 138/37,128 | 1.00 | 156/23,009 | 1.00 |
| 2 | 145/38,187 | 1.02  0.81, 1.30 | 142/23,807 | 0.83  0.66, 1.04 |
| 3 | 130/38,539 | 0.92  0.72, 1.17 | 125/24,032 | 0.72  0.56, 0.91 |
| 4 | 121/39,111 | 0.88  0.68, 1.27 | 123/24,247 | 0.70  0.55, 0.89 |
| 5 | 131/39,594 | 0.96  0.75, 1.24 | 121/24,647 | 0.69  0.54, 0.88 |
| *P-trend across quintiles*^a^ |  | *0.42* |  | *0.001* |
| *P-trend, continuous score*^ab^ |  | *0.63* |  | *0.001* |
|  |  |  |  |  |
| Low-fat products |  |  |  |  |
| 1 | 146/37,925 | 1.00 | 145/23,329 | 1.00 |
| 2 | 132/38,359 | 0.94  0.74, 1.19 | 138/23,733 | 1.02  0.80, 1.29 |
| 3 | 128/38,853 | 0.88  0.70, 1.12 | 125/24,352 | 0.89  0.70, 1.14 |
| 4 | 130/38,361 | 0.91  0.72, 1.16 | 136/23,725 | 0.99  0.78, 1.26 |
| 5 | 129/39,056 | 0.87  0.68, 1.11 | 123/24,602 | 0.87  0.68, 1.11 |
| *P-trend across quintiles*^a^ |  | *0.26* |  | *0.26* |
| *P-trend, continuous score*^ab^ |  | *0.20* |  | *0.37* |
|  |  |  |  |  |
| Dressing and vegetables |  |  |  |  |
| 1 | 192/37,412 | 1.00 | 168/22,622 | 1.00 |
| 2 | 165/38,242 | 1.03  0.74, 1.27 | 143/23,497 | 0.96  0.77, 1.21 |
| 3 | 130/38,910 | 0.96  0.76, 1.20 | 136/23,880 | 0.98  0.78, 1.24 |
| 4 | 100/38,929 | 0.88  0.68, 1.14 | 121/24,633 | 0.97  0.76, 1.25 |
| 5 | 78/39,066 | 0.95  0.71, 1.27 | 99/25,109 | 0.91  0.70, 1.19 |
| *P-trend across quintiles*^a^ |  | *0.38* |  | *0.59* |
| *P-trend, continuous score*^ab^ |  | *0.62* |  | *0.33* |

*^a^*Adjusted for age, season, diet method version, total energy intake, leisure time physical activity, smoking, alcohol intake, education, and baseline BMI

^b^P-trend per unit of the pattern factor

Table 5. Weight change during follow-up in quintiles of data driven dietary patterns in women and men from the Malmö Diet and Cancer cohort.

|  | 10-year weight change^*^ | |
| --- | --- | --- |
| Quintiles of dietary patterns | Women(n=1533) | Men (n=1094) |
| *Health conscious* |  |  |
| 1 | 1.93^a^±1.26 | 2.04^a^ ±1.17 |
| 2 | 2.10^a^±1.27 | 1.65^ab^±1.17 |
| 3 | 2.30^a^±1.25 | 1.12^b^ ±1.17 |
| 4 | 1.96^a^±1.26 | 1.40^b^ ±1.16 |
| 5 | 1.97^a^±1.25 | 1.11^b^ ±1.15 |
| P-trend across quintiles | 0.81 | 0.03 |
| P-trend, continuous score^#^ | 0.89 | 0.09 |
| *Low-fat products* |  |  |
| 1 | 1.77^a^±1.25 | 1.11^a^ ±1.18 |
| 2 | 2.45^a^±1.25 | 1.44^a^ ±1.16 |
| 3 | 2.07^a^±1.26 | 1.42^a^ ±1.16 |
| 4 | 2.12^a^±1.26 | 1.14^a^ ±1.17 |
| 5 | 2.15^a^±1.25 | 1.14^a^ ±1.17 |
| P-trend across quintiles | 0.68 | 0.76 |
| P-trend, continuous score^#^ | 0.91 | 0.78 |
| *Dressing and vegetables* |  |  |
| 1 | 1.96^a^±1.25 | 1.31^a^ ±1.18 |
| 2 | 1.84^a^±1.26 | 1.56^a^ ±1.17 |
| 3 | 1.81^a^±1.25 | 1.28^a^ ±1.16 |
| 4 | 1.90^a^±1.26 | 1.08^a^ ±1.16 |
| 5 | 2.46^b^±1.25 | 1.55^a^  ±1.16 |
| P-trend across quintiles | 0.20 | 0.99 |
| P-trend, continuous score^#^ | 0.16 | 0.93 |

*Homogenous subsets are indicated by letters. Adjusted for age, season, diet method version, total energy intake, leisure time physical activity, smoking, alcohol intake, education, and baseline BMI

# P-trend per unit of the pattern factor
